# MiR-146b accelerates osteoarthritis progression by targeting alpha-2-macroglobulin

**DOI:** 10.18632/aging.102160

**Published:** 2019-08-17

**Authors:** Xin Liu, Liangliang Liu, Hongbo Zhang, Yan Shao, Ziyu Chen, Xiaofeng Feng, Hang Fang, Chang Zhao, Jianying Pan, Haiyan Zhang, Chun Zeng, Daozhang Cai

**Affiliations:** 1 Department of Orthopedics, Academy of Orthopaedics, Guangdong Province, The Third Affiliated Hospital of Southern Medical University, Guangzhou 510630, China; 2 Orthopaedic Hospital of Guangdong Province, Guangzhou 510630, China

**Keywords:** miR-146b, osteoarthritis, alpha-2-macroglobulin, chondrocytes, apoptosis

## Abstract

Osteoarthritis (OA) is an aging-related chronic degenerative disease characterized by the degradation of chondrocyte extracellular matrix (ECM). Previous studies have suggested that microRNAs (miRNAs) are associated with OA, but the role of miR-146b in OA remains unclear. The aim of this study was to determine the role of miR-146b in OA progression. The effect of miR-146b on ECM degradation were studied in mouse chondrocytes transfected with miRNA and treated with IL-1β. Cell viability and the expression levels of proteolytic enzymes in the transfected cells were assessed by real-time RT-PCR, ELISA and Western blots. We found downregulation of miR-146b expression in chondrocytes dramatically inhibited IL-1β-induced caspase activation and proteolytic enzyme expression via influencing its targeted Alpha-2-macroglobulin (A_2_M). Luciferase reporter assays confirmed that A_2_M mRNA was negatively regulated by miR-146b in chondrocytes. Intra-articular injection of antago-miR-146b against miR-146b effectively protected mice from the progression of DMM-induced osteoarthritis by inhibiting cartilage proteoglycan degradation. Our study indicates that miR-146b plays a critical role in the progression of injury-induced osteoarthritis by directly targeting A_2_M expression to elevate the proteolytic enzyme production and stimulate chondrocytes apoptosis, and miR-146b as well as A_2_M could be therapeutic targets.

## INTRODUCTION

Osteoarthritis (OA) is the most common degenerative disease caused by joint instability in the elderly [1, 2]. The main characteristic of OA includes progressive degeneration of articular cartilage, subchondral bone remodeling and joint inflammation that ultimately lead to severe joint pain and loss of function [3, 4]. Its pathogenesis of OA is not well understood and there is no curable medical therapy for this disease. Chondrocytes in cartilage function normally produce and secret the extracellular matrix proteins to maintain cartilage integrity [5]. Under the disease conditions, however, the chondrocytes change their behavior, they become autophagy, overexpress the hypertrophy markers, and secrete disease-causal cytokines or small fragments of nucleic acid—such as microRNAs (miRNAs) to promote cell degeneration and apoptosis [6, 7].

MiRNAs are a class of small, single-stranded, non-coding RNA that can bind to complementary target sequences of the 3′ untranslated regions (3′-UTR) of mRNAs to regulate mRNA stability and translation [8, 9]. Previous studies have demonstrated that the alteration in the expression of specific miRNAs [7] are associated with cartilage damage and OA development. MiRNAs, including miR-101 [10], miR-675 [11], miR-140 [12], miR-27a [13], miR-127-5P [14], miR-15a [15], and miR-130a [16] have been reported to be involved in various processes of OA pathogenesis by regulation of cartilage homeostasis, chondrocyte metabolism, proteolytic enzyme activity, and inflammatory responses. These studies suggest that miRNA expression could play a pivotal role in the OA development and progression. Therefore, identifying the miRNA regulatory network involved in OA and understanding the mechanisms are needed to development novel therapeutic strategies for OA prevention and treatment. MiR-146b has been reported have multiple functions including regulation of cell differentiation, proliferation, and migration in various tumor cells [17, 18]. Recent studies have observed that miR-146b is over-expressed in the articular chondrocytes in the osteoarthritic tissues [19]. However, the role of miR-146b in OA progression, and the mechanism by which miR-146b controls the fate of chondrocytes are not known.

Inflammatory cytokines have been known to be involved in the development and progression of OA [20]. Among them, interleukin-1β (IL-1β) was dramatically up-regulated in chondrocytes of the superficial and middle layers of the cartilage during OA development and progression [21]. Treatment of IL-1β stimulated the ECM-degrading enzyme syntheses and secretion, leading to the chondrocyte ECM breakdown and OA development [22], while suppression of IL-1β-induced inflammatory factors with neutralizing antibodies or small molecular inhibitors was able to prevent and treat OA [23, 24]. These studies indicate that IL-1β is one of the major inflammatory factors in OA. In this study, we investigated the effects of miR-146b on the ECM-degrading enzyme syntheses and secretion in IL-1β-treated chondrocytes *in vitro*, explored its potential target gene, and examined the role of miR-146b in ECM degradation and OA progression in destabilization of the medial meniscus (DMM)-induced OA in mice. Our findings provide direct evidence for the role of miR-146b in OA and support the hypothesis that inhibiting the endogenous activity of miR-146b could be an attractive approach to prevent and treat OA.

## RESULTS

### MiR-146b is overexpressed in the OA cartilage and IL-1β-treated chondrocytes

**Figure 1:**
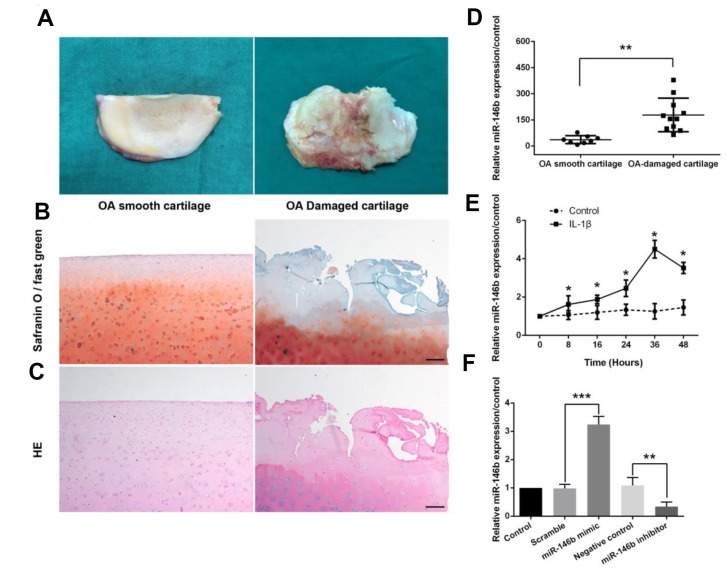
**miR-146b expression is up-regulated in OA-damaged cartilage tissues and in IL-1β-stimulated mouse chondrocytes.** (**A**) Representative macromorphological images of smooth cartilage and damaged cartilage from OA patients. (**B**, **C**) Safranin O-fast green and H&E staining of smooth OA cartilage and damaged OA cartilage. Scale bar: 100μm. (**D**) The expression level of miR-146b in smooth and damaged cartilage tissues. (**E**) Time course of the miR-146b expression under IL-1β stimulation in mouse chondrocytes. (**F**) mRNA level of miR-146b in mouse chondrocytes transfected with miR-146b-mimic and miR-146b-inhibitor were evaluated by qRT-PCR. The negative control of miR-146b mimic and miR-146b inhibitor were referred as to scramble and NC. *P<0.05, **P<0.01, ***P<0.001.

To investigate the potential role of miR-146b in OA, we measured the expression levels of miR146b in the cartilage and IL-1β-treated chondrocytes by qRT-PCR. The OA-damaged cartilage and smooth cartilage of the human knee from the arthroplasty surgery were dissected and stained with Safranin O-fast green and HE, respectively (Figure 1A–1C). We found that articular cartilage was severely damaged in OA-damaged cartilage, and the level of miR-146b expression was 6.72-fold higher in OA-damaged cartilage as compared to the smooth tissues (Figure 1D). To furthermore examine the expression level of miR-146b in chondrocytes, we cultured the primary chondrocytes from mice, and treated with IL-1β or vehicle. We found that IL-1β treatment of mouse chondrocytes increased miR-146b expression in a time-dependent manner, with highest expression of 4.28-fold over the vehicle control 36 hours after treatment (Figure 1E). The increased expression of miR-146b in OA indicated that miR-146b may contribute to its pathogenesis. Thus, we transfected chondrocytes with miR-146b inhibitor or miR-146b mimic to assess the effect of miR-146b on IL-1β-induced apoptosis and cartilage matrix degradation. As expected, the expression level of miR-146b was elevated by 3.31-fold in the cells transfected with miR-146b mimic compared to the cells transfected with a scramble control RNA. By contrast, transfection of chondrocytes with miR-146b inhibitor destabilized miR-146b and caused a 69% decrease in miR146b level (Figure 1F).

### MiR-146b promotes IL-1β-induced apoptosis and extracellular matrix degradation in chondrocytes

**Figure 2:**
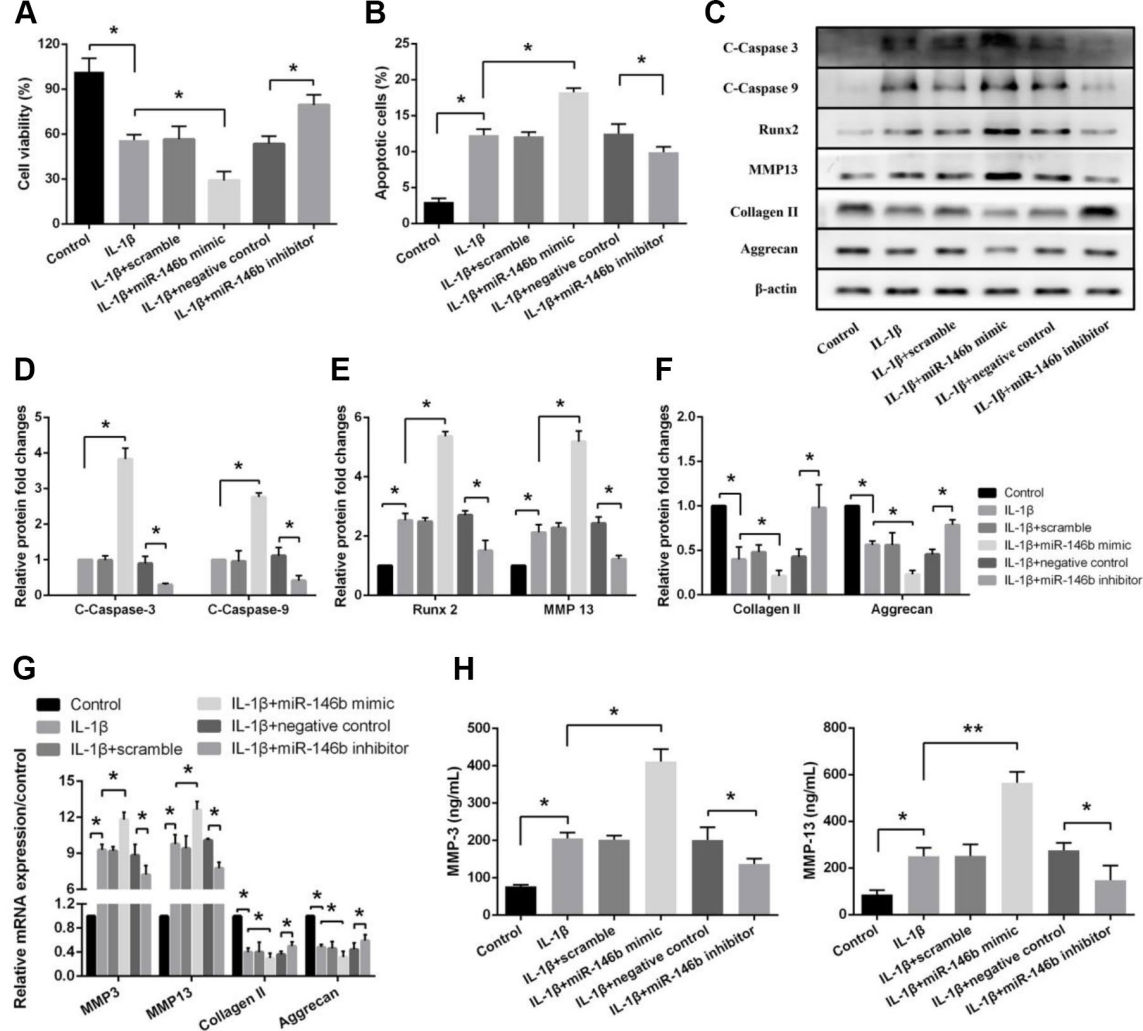
**miR-146b promotes IL-1β-induced apoptosis and extracellular matrix degradation in chondrocytes**. (**A**) Cell viability, (**B**) Cell apoptosis and (**C**–**F**) The expression of apoptosis-related and catabolic event proteins were detected by CCK8 assay, flow cytometry and western blot. (**G**) mRNA levels of chondrocytes catabolic event markers (MMP3 and MMP13), articular chondrocyte marker (Collagen II and Aggrecan) were tested by qRT-PCR. The concentrations of (**H**) MMP3 and MMP13 in the culture supernatant were measured by ELISA. *P<0.05, **P<0.01.

To determine if the increased expression level of miR-146b in chondrocytes contributes to the cell death and matrix degrading enzyme production, we measured the cell viability and the enzyme expression levels in IL-1β-treated cells. We found that boosted level of miR-146b in chondrocytes remarkably decreased IL-1β-induced cell viability by 48% and increased IL-1β-stimulated cell apoptosis by 43%, while transfection of chondrocytes with miR-146b inhibitor increased cell viability by 39% and decreased apoptosis by 21% (Figure 2A, 2B). Consistent with the cell viability, the expression of apoptosis-related factors of C-Caspase-3 and C-Caspase-9 that are involved in caspase activation and the expression of hypertrophic factors, Runx2 and MMP13 were significantly elevated in the chondrocytes transfected with miR-146b, whereas the expression of chondrocyte markers, collagen II and aggrecan were decreased by 47% and 59%, respectively. In addition, the expression of catabolic factors were also increased significantly in the cells transfected with miR-146b mimic compared with the control cells (Figure 2C–2G, Supplementary Figure 1). In contrast, transfection of the cells with miR-146b inhibitor caused decreased expression of C-Caspase-3 and C-Caspase-9 as well as hypertrophic factors of Runx2 and MMP13 but increased expression of collagen II and aggrecan. To confirm our Western blot data, we also measured the protein levels of MMP3 and MMP13 in cell culture supernatant by ELISA. Similarly, we found that the expression of MMP3 and MMP13 were increased 2.27-fold and 2.76-fold respectively in miR-146b mimic-transfected cells (Figure 2H). Taken together, these data indicated that the overexpression of miR-146b promoted apoptosis and cartilage matrix degradation induced by IL-1β in chondrocytes.

### MiR-146b negatively regulates the expression of A_2_M in chondrocytes

**Figure 3:**
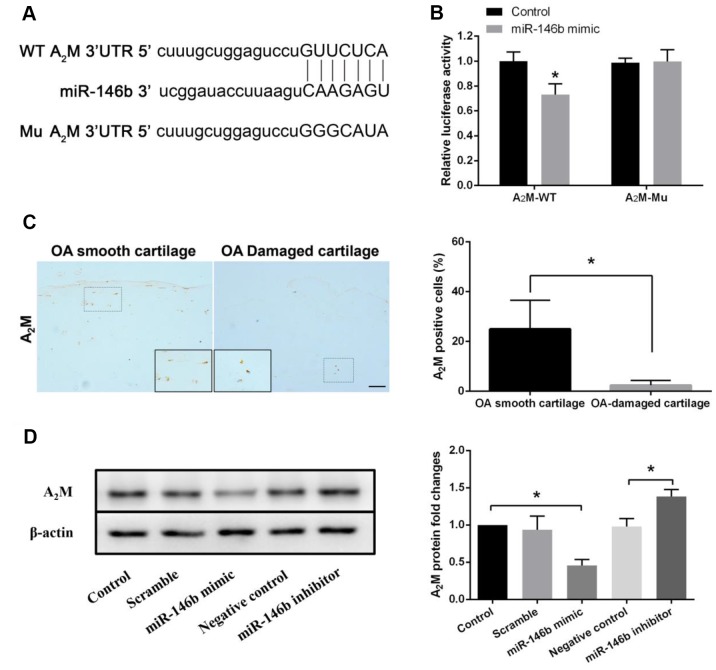
**miR-146b negatively regulates the expression of A_2_M.** (**A**) miR-146b aligned with the 3**′**UTR of A_2_M mRNA. (**B**) chondrocytes co-transfected with a reporter carrying the wild type (wt) A_2_M 3′UTR along with miR-146b mimics. The A_2_M mutant (mu) recombinant vector was used as positive control. Targeting effect was measured by luciferase assay. (**C**) Immunohistochemical analysis of A_2_M expression in smooth OA cartilage and damaged OA cartilage. Scale bar: 100μm. The protein (**D**) levels of A_2_M expression in chondrocytes transfected with miR-146b-mimic and miR-146b-inhibitor were evaluated by western blot. *P<0.05.

To further investigate the potential molecular mechanisms of miR-146b in chondrocytes, we screened the putative target genes of miR-146b through bioinformatic analyses. Among the candidate target genes, we found that A_2_M, a major protease inhibitor secreted by hepatocytes, was a direct target of miR-146b (Figure 3A). To verify whether miR-146b bound directly to its target sequence in the 3′-UTR of A_2_M mRNA in chondrocytes, we constructed luciferase reporters in which the wild type (WT) or mutated (Mu) A_2_M 3′-UTR that miR-146b binds was fused to the luciferase gene. As shown in Figure 3B, miR-146b effectively inhibited luciferase activity in chondrocytes expressing the luciferase reporter containing WT 3′-UTR of A_2_M but not the reporter bearing the Mu A_2_M 3′-UTR compared to the control reporter. In agreement with the reporter assays, we detected a 78% decrease in A_2_M expression level in the OA-damaged cartilage as compared to the smooth cartilage (Figure 3C). In addition, we observed that overexpression of miR-146b reduced A_2_M protein expression by 53% in chondrocytes while transfection of the chondrocytes with the miR-146b inhibitor increased A_2_M protein expression by 41%, as detected by Western blot analyses (Figure 3D). These results suggest that miR-146b negatively regulates the expression of A_2_M.

### Suppression of miR-146b function inhibits IL-1β-induced apoptosis and extracellular matrix degradation in chondrocytes via upregulating A_2_M expression

**Figure 4:**
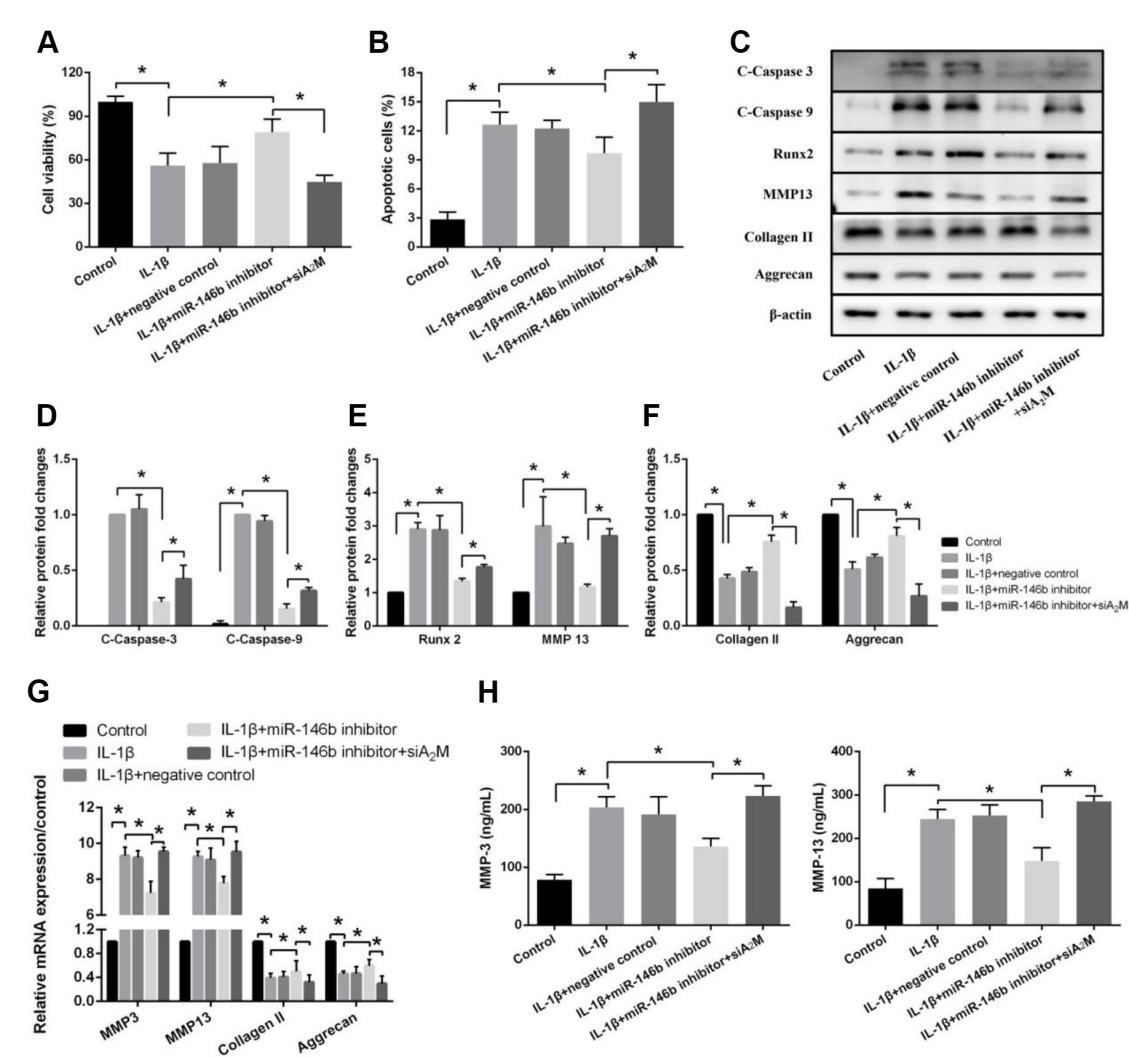
**Suppression of miR-146b inhibits IL-1β-induced apoptosis and extracellular matrix degradation in chondrocytes by upregulating A_2_M expression.** (**A**) Cell viability, (**B**) Cell apoptosis and (**C**–**F**) The expression of apoptosis-related and catabolic event proteins were detected by CCK8 assay, flow cytometry and western blot. (**G**) mRNA levels of chondrocytes catabolic event markers (MMP3 and MMP13), articular chondrocyte marker (Collagen II and Aggrecan) were tested by qRT-PCR. The concentrations of (**H**) MMP3 and MMP13 in the culture supernatant were measured by ELISA. *P<0.05.

To examine the effects of A_2_M on IL-1β-induced chondrocyte apoptosis and extracellular matrix degradation. Chondrocytes were transfected with siA_2_M to knock down the A_2_M expression (Supplementary Figure 3). We observed marked increases in viability in cells transfected with IL-1β+miR-146b inhibitor compared with the other groups (Figure 4A). We also observed a significant decrease in the percentage of apoptotic cells in the IL-1β+miR-146b inhibitor group compared with the IL-1β+NC group (Figure 4B). However, after chondrocytes were co-transfected with siA_2_M, the relative number of apoptotic cells increased compared with the miR-146b inhibitor group. Similar results were obtained by Western blotting analyses (Figure 4C–4F, Supplementary Figure 2). Co-transfection of chondrocytes with siA_2_M increased the expression of C-Caspase-3 by 1.98-fold, C-Caspase-9 by 2.07-fold, Runx2 by 1.32-fold, and MMP13 by 2.31-fold while transfection of the cells with miR-146b inhibitor decreased the expression of these marker genes. Consistent with the Western blot analyses, we found the similar changes in the expression levels of the marker genes by qRT-PCR assays (Figure 4G). As expected, the expression levels of MMP3 and MMP13 were lower in the IL-1β+miR-146b inhibitor group than in the IL-1β+NC group, while both were significantly elevated after co-transfection with siA_2_M (Figure 4H). Collectively, these results indicated that miR-146b induces apoptosis and cartilage matrix degradation in chondrocytes by regulating the expression of A_2_M.

### Inhibition of miR-146b in cartilage delays the progression of osteoarthritis in mice

**Figure 5:**
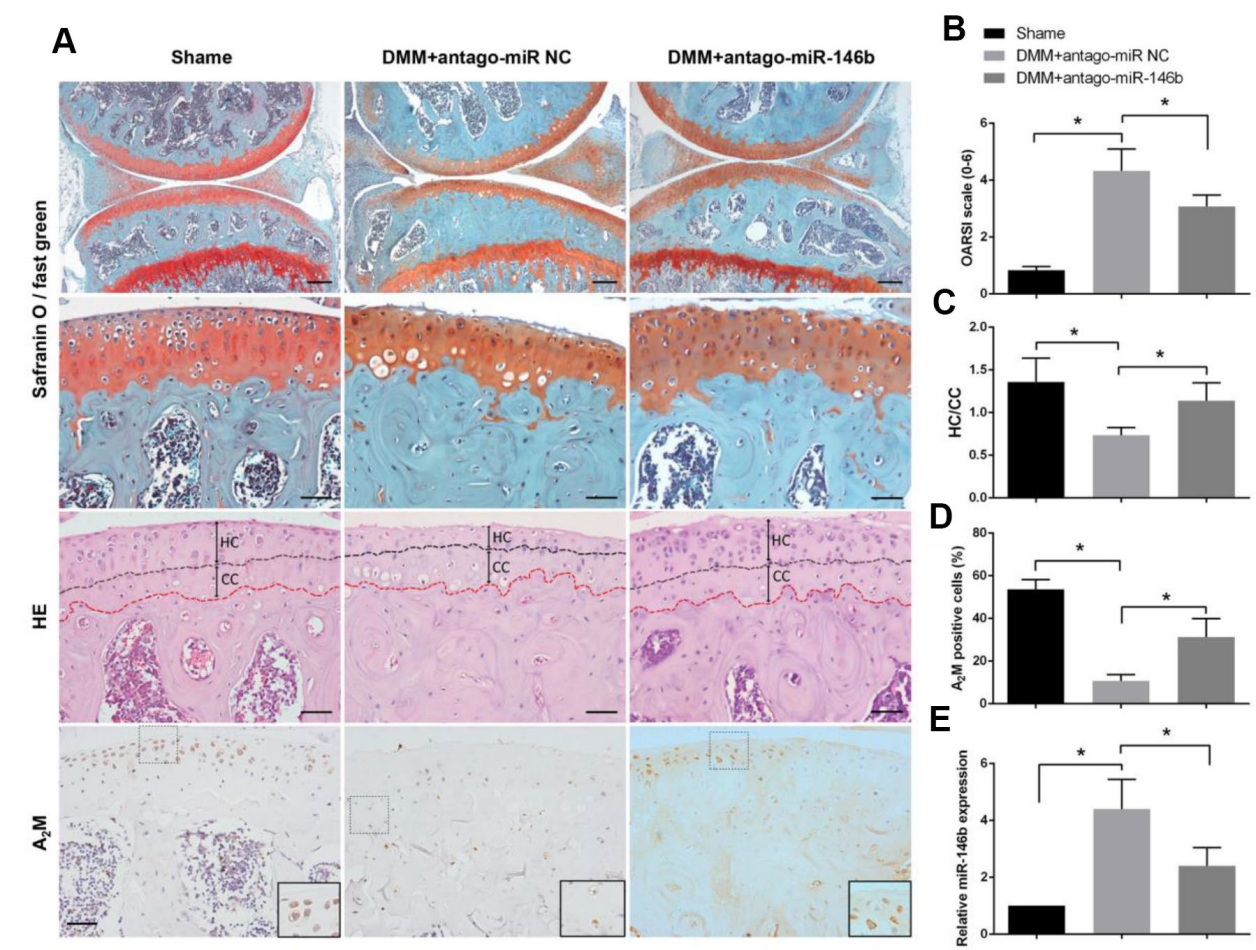
**Inhibition of miR-146b in cartilage delays the progression of osteoarthritis in mice.** (**A**) After DMM surgery or sham operation, mice were intra-articularly injected with 250 μM antago-miR-146b or antago-miR NC on day 7 and day 14 after surgery. Knee joints were harvested at 5 weeks after last injection and analysed histologically by Safranin O-fast green staining. (**B**) OARSI scores based on safranin O and fast green staining in (**A**). (**C**) Quantification of HC/CC according to H&E staining in (**A**). (**D**) Immunostaining analysis of positive A_2_M. (**E**) qRT-PCR analysis of miR-146b levels in mice knee joint cartilage. Scale bar: 100μm (first line); 50μm (others). *P<0.05.

To determine the role of miR-146b in OA progression *in vivo*, a surgical OA model were established in mice, followed by intra-articular injection of antago-miR-146b or antago-miR-NC. Histologic examination revealed that antago-miR-146b protected the cartilage from degradation and decreased the amount of fibrous cartilage. The OARSI scores were significantly lower in the antago-miR-146b-treated mice than in the antago-miR-NC-injected mice (Figure 5A, 5B). As shown in Figure 5C, distance of the tidemark to the articular surface was significantly increased in mice injected with antago-miR-146b as compared to the mice injected with antago-miR-NC. The expression level of miR-146b reduced by 48% in the articular cartilage of the mice injected with antago-miR-146b as compared the mice injected with antago-miR-NC at 5 weeks post operation (Figure 5E). Consistent with the role of miR-146b in chondrocytes, the expression levels of A_2_M was increased 2.3 fold in the articular tissue of the mice injected with antago-miR-146b as compared to the mice injected with antago-miR-NC (Figure 5A, 5D).

**Figure 6:**
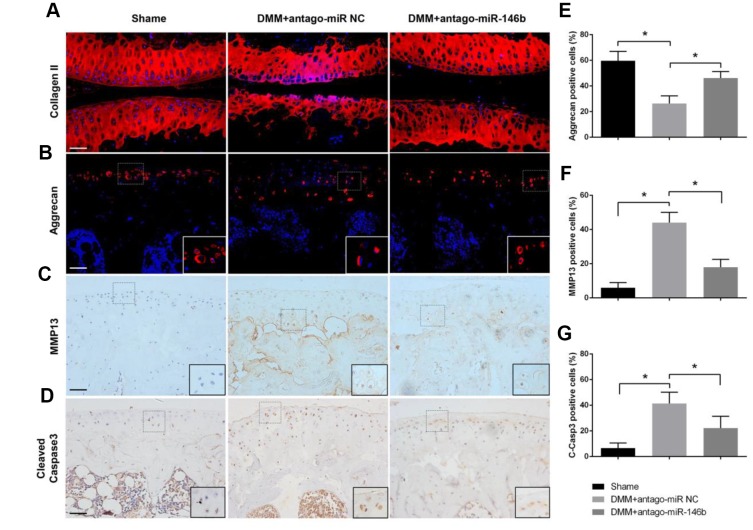
**Effects of miR-146b on the homoeostasis of articular cartilage**. Sections of articular cartilage from the mice were analysed by Immunostaining. Immunofluorescence stainings of (**A**) Collagen II, (**B**) Aggrecan. Immunohistochemical stainings of (**C**) MMP13 and (**D**) Cleaved Caspase-3. (**E**–**G**) The ratios of immunoreactive positive cells, Aggrecan (**E**), MMP13 (**F**) and Cleaved Caspase-3 (**G**) were analysed. Scale bar: 50μm. *P<0.05.

To analyses the effect of miR-146b on the catabolic activities of cartilage, we examined the pathological changes in articular cartilage. MMP13 expression was decreased by 51% while the expression levels of collagen II and aggrecan were increased in antago-miR-146b group compared to antago-miR-NC group (Figure 6A–6F). In addition, antago-miR-146b treatment significantly decreased the expression of C-Caspase-3, an indicator of apoptosis associated with OA progression, by 42% (Figure 6D, 6G) [25]. Collectively, these data suggested that there are therapeutic effects of inhibiting miR-146b on delaying cartilage damage during OA.

### Effects of miR-146b on PI3K/AKT signaling in IL-1β-induced chondrocytes

**Figure 7:**
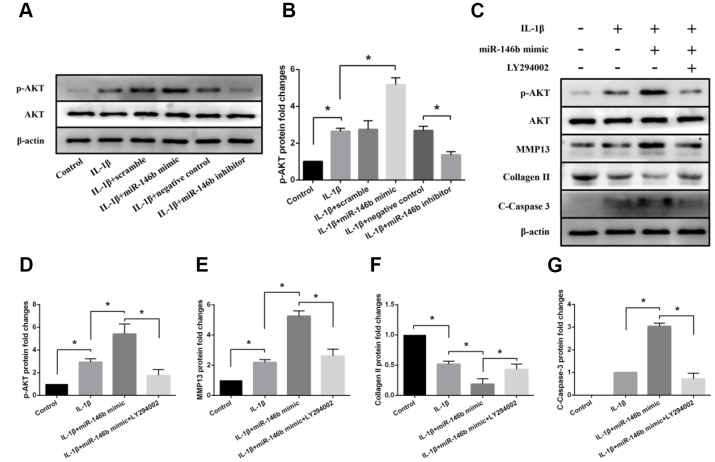
**Effects of miR-146b on PI3K/AKT signalling in IL-1β-induced chondrocytes.** (**A**, **B**) Representative western blots and quantification data of p-Akt and Akt in each group. (**C**–**G**) Representative western blots and quantification data of p-Akt, Akt, MMP13, Collagen II, and Cleaved Caspase 3 in each group. *P<0.05.

To investigate the mechanisms by which inhibition of miR-146b function in chondrocytes alleviated IL-1β-induced cell apoptosis and cartilage matrix degradation in the cartilage, we performed western blot analyses for the signal molecules involved in PI3K/AKT signaling pathways. We found that increased expression level of miR-146b in chondrocytes activated PI3K/ AKT signaling as the phosphorylation at Ser473 of AKT, an active form of AKT, was elevated. As shown in Figure 7A and 7B, transfection of chondrocytes with miR-146b mimic caused a 1.96-fold increase the phosphorylation of AKT in the cells treated with IL-1β. By contrast, treatment of chondrocytes with miR-146b inhibitor decreased AKT phosphorylation by 51% while the total amounts of AKT proteins in the treated cells were not changed.

To examine whether the effects of miR-146b on chondrocytes were modulated by PI3K/ AKT signal pathway, we treated the chondrocytes with LY294002, a specific PI3K inhibitor in the presence of IL-1β. Western blot analyses revealed that LY294002 treatment of miR-146b mimic transfected chondrocytes decreased MMP13 expression by 52% and increased collagen II expression by 2.31-fold (Figure 7C–7F). In addition, LY294002 treatment abolished miR-146b induced chondrocyte apoptosis, as the apoptotic marker C-Caspase-3 was reduced in the miR-146b transfected cells treated with LY294002 (Figure 7C, 7G). Together, these results suggest that PI3K/AKT signaling was involved in the regulation of miR-146b on chondrocyte metabolism.

## DISCUSSION

MiRNAs are a class of endogenous small non-coding RNAs of 18-22 nucleotides in length. The major role of miRNAs is to regulate target genes via translational inhibition and/or target mRNA degradation. Accumulating evidence shows that certain miRNAs could directly target distinct genes involved in the pathogenesis and development of OA and play vital roles in regulating the activity and function of chondrocytes. For example, Makki MS et al. showed that miR-9 directly targeted the “seed-sequence” of the MCPIP1 3′UTR, resulting in its downregulation and an increase in IL-6 expression in human OA chondrocytes [26]. Our previous studies also showed that miR-483-5p directly targeted the cartilage matrix protein matrilin 3 and tissue inhibitor of metalloproteinase 2 to stimulate chondrocyte hypertrophy, extracellular matrix degradation, and cartilage angiogenesis as such initiate the development of OA [27]. In this study, we demonstrated that miR-146b was upregulated in IL-1β-treated chondrocytes. Through gain-of-function and loss-of-function studies, we found that miR-146b significantly affected cell viability and matrix gene expressions in chondrocytes. The downregulation of miR-146b dramatically inhibited caspase activation and the expression of proteolytic enzymes. More importantly, intra-articular injection of antago-miR-146b effectively protected mice with OA from cartilage degradation and proteoglycan loss compared with control mice. Our findings suggest that upregulation of miR-146b might contribute to the development and progression of OA. Thus, inhibition of endogenous miR-146b function could be an alternative therapeutic approach for OA prevention and treatment.

miR-146b has been associated with development and progress of the tumors such as hepatocellular carcinoma [28], human gliomas [29], and papillary thyroid carcinoma [30]. Previous studies on miR-146b were mainly focused on its overexpression in tumor tissues and its inhibition of apoptosis and promotion of the cancer progression. In a recent study reported by Budd et al., miR-146b was identified as a critical regulator of chondrogenic differentiation from the human bone marrow-derived skeletal stem cells, as such it may play an important role in the pathogenesis of OA development [19]. Authors found that the elevated miR-146b expression was accompanied by the down-regulation of SOX5 in skeletal stem cells. SOX5, regulates genes responsible for cartilage production and cartilage ECM formation during the early chondrogenic differentiation [31, 32]. Consistent with previous studies, we found that overexpression of miR-146b decreased the expression of the ECM proteins, collagen II and aggrecan but enhanced the expression of the ECM-degrading enzymes, MMP-3 and MMP-13. In addition, we identified A_2_M as one of miR-146b targets in OA development. A_2_M, a major protease inhibitor, is produced mainly in hepatocytes. Recent studies have indicated that A_2_M, in addition to its role as a protease inhibitor, could be involved in maintaining the dynamic equilibrium of the cartilage ECM microenvironment [33, 34]. After bovine cartilage explants were incubated with TNF-α and IL-1β, treatment with A_2_M variants significantly inhibited catabolism and decreased proteolytic enzyme activity [35]. Furthermore, intra-articular supplementation with A_2_M had a profound chondroprotective effect in a rat model of OA through reducing the concentration of MMP-13 in synovial fluid [36]. In this study, we demonstrated that miR-146b could directly target A_2_M to disrupt the balance of the cartilage ECM microenvironment. Knockdown of A_2_M significantly impaired the antiapoptosis and cartilage-protection of a miR-146b antagonist, confirming that A_2_M was the direct target of miR-146b to suppress cartilage degeneration, proteoglycan loss, and chondrocyte apoptosis. Unraveling the role of miR-146b in joint physiology and pathology will shed light on the prevention and treatment of OA.

Based on these findings, we propose that miR-146b suppression may contribute to antiapoptotic and protect chondrocyte from extracellular matrix degradation during the development and progression of OA by up-regulating A_2_M expression. Our findings provide a theoretical basis for better understanding of miR-146b function on OA and imply that miR-146b may be a potential target of intervention for the prevention of OA.

## MATERIALS AND METHODS

### Human cartilage specimens

OA patients were diagnosed according to the American College of Rheumatology criteria. Articular cartilage tissues were collected from 19 patients who underwent knee arthroplasty surgery (12 women and 7 men; age (mean ± SEM) 65.28 ± 4.07 years). After washing with sterile phosphate-buffered saline (PBS) buffer, portions of cartilage with damaged articular surface and portions with smooth articular surface were used for histological staining, RNA extraction, and immunostaining. Human study was approved by the Ethics Committee of the third Affiliated Hospital of Southern Medical University, and all samples were collected after informed consent.

### Experimental osteoarthritis mouse model

C57BL/6J WT mice were purchased from the Laboratory Animal Centre of Southern Medical University. All mice were housed in a pathogen-free animal facility at the university. Experimental OA was surgically induced in the right knee according to the protocol for DMM surgery has been published by our lab previously [37]. In the DMM-induced group, the right medial collateral ligaments and anterior cruciate ligaments were dissected, followed by transection of the medial meniscus in the right knee. In the sham-operated group, only the skin of the right knee joint was resected. Animals were sacrificed at 5 weeks after knee surgery for collection of knee joint specimens. All animal experiments were approved by the Southern Medical University Committee on the Use and Care of Animals and were performed in accordance with the committee’s guidelines.

### Intra-articular Injection

For the antago-miR-146b injection, 250 μM antago-miR-146b (AGCCUAUGGAAUUCAGUUCUCA) or antago-mir NC (GenePharma, Shanghai, China) were injected into the knee joint [38] of male mice (n = 5/group) using a 33G needle and a micro-syringe (Hamilton, Bonaduz, GR, Switzerland). All experimental mice were injected on day 7 and day 14 after surgery in the OA model. Knee joints were harvested 5 weeks later.

### Cell culture and treatment

Primary chondrocytes was obtained from rib cartilage of newborn mice as previously described [39]. After isolation, cells were cultured in 75cm^2^ culture flasks with growth media (DMEM/F12 containing 10% fetal bovine serum, 100 U/ml penicillin and 100 mg/ml streptomycin; Sigma-Aldrich). Non-adherent cells were removed, and adherent chondrocytes were cultured and expanded for further experiments. Primary cells were used in the experiments prior to the third passage. Cells were cultured with IL-1β (Sigma-Aldrich, St. Louis, Mo, USA) at the concentration of 10ng/ml.

### Cell viability assay

Primary chondrocytes were seeded in 96-well plates at a density of 6 × 10^3^ cells/well. After 12h in culture, cells were treated with IL-1β for 24h. Cell viability was assessed using the Cell Counting Kit-8 (CCK-8; Keygen Biotech, Nanjing, China) according to the manufacturer’s instructions. Absorbance was measured at 450 nm.

### Apoptotic assay

Chondrocytes were seeded into 6-well plates at a density of 1 × 10^5^ cells per well. Cell apoptosis rates were evaluated by flow cytometry using the Annexin V/PI apoptosis detection kit (BD Biosciences, Franklin Lakes, NJ, USA). Chondrocytes were washed twice cold PBS, and then resuspended in binding buffer and incubated with 5μl FITC-Annexin V and 5μl PI at room temperature for 15 min in the dark. Subsequently, staining cells were determined by using the FACScan flow cytometry system (Becton Dickinson, San Diego, CA, USA).

### Enzyme-linked immunosorbent assay (ELISA) assay

Following treatment of IL-1β for 24 h as described above, the levels of MMP3, and MMP13 in the culture medium supernatant were evaluated by ELISA kits (R & D Systems, Minneapolis, USA) according to the protocols from the manufacturer.

### miRNAs transfection

miR-146b mimic, miR-146b inhibitor, siRNA directly against A_2_M (si-A_2_M) and the corresponding negative controls (NC) were synthesized by GenePharma (Shanghai, China). Cell transfections were conducted by using Lipofectamine 3000 reagent (Invitrogen, Carlsbad, USA) according to the manufacturer’s instructions protocol. The sequences for miR-146b mimic and inhibitor were as follows: mimic sense, UGAGAACUG AAUUCCAUAGGCU, mimic anti-sense, CCUAUGGA AUUCAGUUCUCAUU, inhibitor, AGCCUAUGGAAU UCAGUUCUCA.

### Bioinformatics analysis

Online tools including miRanda (http://www.microrna.org/microrna/home.do), RNAhybrid (http://bibiserv.techfak.uni-bielefeld.de/rnahybrid/) and Targetscan (http://www.targetscan.org/vert_71/) were used to predict the targets of miR-146b.

### Dual luciferase activity assay

The 3′UTR target site as well as the luciferase reporter constructs with the A_2_M 3′UTR carrying the putative miR-146b-binding site into pMiR-report vector were amplified by PCR. Cells were co-transfected with the reporter constructs, control vector and miR-146b or scramble using Lipofectamine 3000 regent (Invitrogen, Carlsbad, USA) following the manufacturer’s instructions. Luminescent signals were quantified by a luminometer (Glomax, Promega).

### Quantitative reverse transcription PCR (qRT-PCR) assay

Total RNA was isolated from chondrocytes cultures using the TRIzol reagent (Life Technologies, Grand Island, NY, USA) according to the manufacturer’s protocol. Then, the total RNA products were immediately transcribed by reverse transcription (RT) into cDNA by using a PrimeScript RT reagent Kit with gDNA Eraser (TaKaRa, Dalian, China). Polymerase chainreaction (PCR) amplification was performed in a Chromo4 Four-Color Real-Time PCR Detection System (Bio-Rad) by using the SYBRR Premix Ex Taq II kit (TaKaRa). Primer sequences (Life Technologies) for each gene used in this study are shown in Table 1.

**Table 1:** Primer sequences used in qRT-PCR.

**Target gene**	**Forward primer**	**Reverse primer**
MMP-3	5′-TGGCATTCAGTCCCTCTATGG-3′	5′-AGGACAAAGCAGGATCACAGTT-3′
MMP-13	5′-AAGGAGCATGGCGACTTCT-3′	5′-TGGCCCAGGAGGAAAAGC-3′
Aggrecan	5′-AGGCAGGGTGATCCTTACC-3′	5′-GGCCTCTCCAGTCTCATTCTC-3′
Collagen-II	5′-ATCTACCGTGAAGCTGATTC-3′	5′-TAGAAGGACGGAACAATTCC-3′
miR-146b	5′-TGACCCATCCTGGGCCTCAA-3′	5′-CCAGTGGGCAAGATGTGGGCC-3′
A_2_M	5′-GGAGACATATTAGGCTCTGC-3′	5′-CTGAAACCTACTGGAAATCC-3′
U6	5′-GCTTCGGCAGCACATATACTAAAAT-3′	5′-CGCTTCACGAATTTGCGTGTCAT-3′
GAPDH	5′-GGCTCTCTGCTCCTCCTGTT-3′	5′-CCATGGTGTCTGAGCGATGT-3′

### Western blot (WB) assay

Lysis buffer was prepared with 10% glycerol, 2% sodium dodecyl sulfate, 10 mM dithiothreitol, 10 mM Tris–HCl (pH 6.8), 1 mM phenylmethylsulfonyl fluoride and 10% β-mercaptoethanol. Cells were lysed by lysis buffer at 98°C for 10 min. Samples were separated by SDS-PAGE for 90 min. Samples were blotted onto nitrocellulose membranes for 1 h and incubated with primary antibodies (in 5% BSA, 0.2% NaN3) at 4°C overnight. Samples were incubated with secondary antibodies at 37°C for 1 h. Western blot assay was performed using monoclonal primary antibodies: Cleaved Caspase-3 (#9661, Cell Signaling Technology, CST), Cleaved Caspase-9 (#9509, CST), Runx2 (A2851, ABclonal), MMP13 (ab84594, Abcam), Collagen II (ab34712, Abcam), Aggrecan (ab3773, Abcam), A_2_M (ab58703, Abcam), AKT (#2920, CST), phosphorylated (p)-AKT (#4060, CST) and β-actin (20536-1-AP, Proteintech).

### Histological and Immunostaining assay

The knee joints from mice were fixed with 4% paraformaldehyde for 24 h and then decalcified with 0.5 M ethylenediaminetetraacetic acid (EDTA) at pH 7.4 for 21 days. The specimens were embedded in paraffin and sectioned at 4 μm. For histological analysis, the samples were stained with hematoxylin and eosin (H&E) and safranin O-Fast Green staining (Sigma-Aldrich). For the immunohistochemistry analysis, we employed the following primary antibodies: A_2_M (ab58703, Abcam), MMP13 (ab84594, Abcam), and Cleaved Caspase-3 (#9661, CST). Sections were then stained with horseradish peroxidase (HRP)-conjugated secondary antibodies (Jackson ImmunoResearch Laboratories, West Grove, PA). For immunofluorescence, the primary antibodies used were Collagen II (ab34712, Abcam), Aggrecan (ab3773, Abcam) and Alexa 594 dye-labeled secondary antibodies (Jackson ImmunoResearch Laboratories, Inc., West Grove, PA). The sections were mounted with medium containing DAPI and images were obtained using a FluoView FV1000 confocal microscope (Olympus, Tokyo, Japan).

### Statistical analysis

All experiments were performed in triplicate. Numerical data are presented as mean ± SD by SPSS version 19.0 software. Two-way analysis of variance (ANOVA) followed by Student’s t-test for two groups or Bonferronitest for more than two groups were used to analyse statistical differences. *p* < 0.05 was considered statistically significant.

## Supplementary Material

Supplementary Figures
