# Supplementary material for: MiR-146b accelerates osteoarthritis progression by targeting alpha-2-macroglobulin
Source: Aging (Albany NY). 2019 Aug 17;11(16):6014–28. doi: 10.18632/aging.102160 (PMC6738400; doi:10.18632/aging.102160)
Supplement: Supplementary Figures [file aging-11-102160-s001.pdf]

SUPPLEMENTARY FIGURES

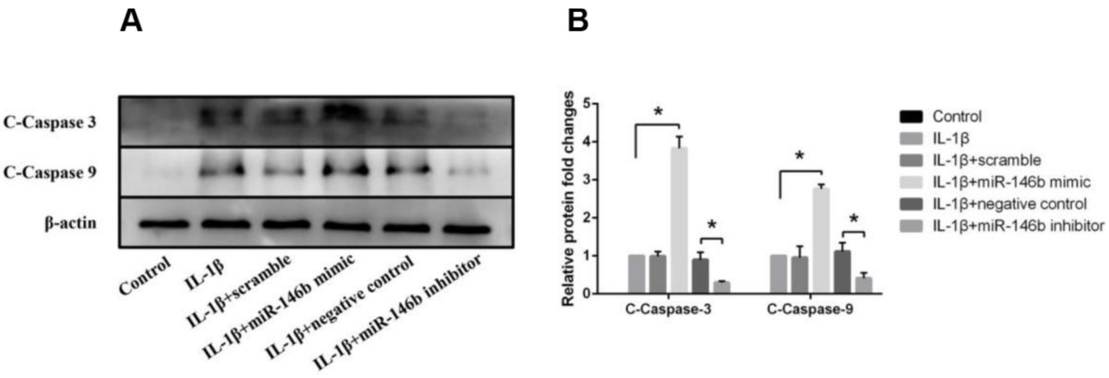

Supplementary Figure 1. miR-146b promotes IL-1β-induced apoptosis in chondrocytes. Chondrocytes were transfected with miR-146b-mimic or miR-146b inhibitor and their negative control. (**A**, **B**) Representative western blots and quantification data of Cleaved Caspase 3 and Cleaved Caspase 9 in each group. **P*<0.05.

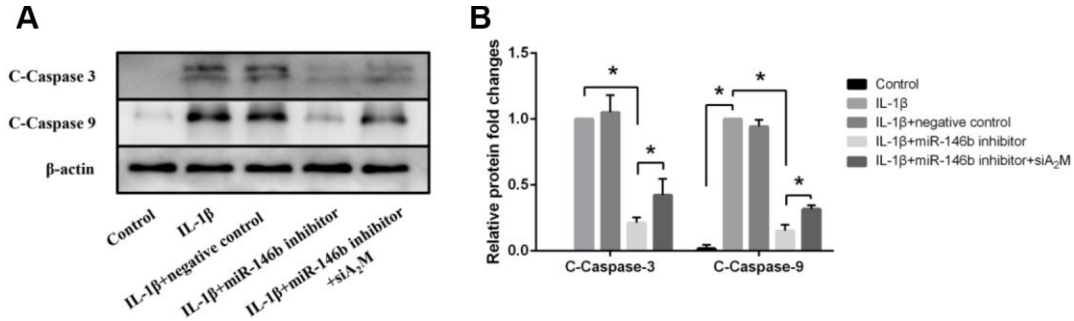

Supplementary Figure 2. Suppression of miR-146b inhibites IL-1β-induced apoptosis in chondrocytes by upregulating A_2M expression. Chondrocytes were transfected with miR-146b inhibitor or co-transfected with miR-146b inhibitor and siA_2M. (**A**, **B**) Representative western blots and quantification data of Cleaved Caspase 3 and Cleaved Caspase 9 in each group. **P*<0.05.

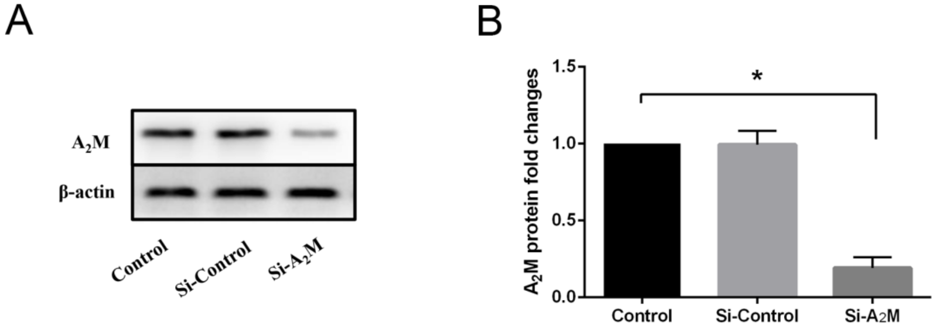

Supplementary Figure 3. Determination of A_2M protein expression by Western blotting in chondrocytes. Chondrocytes were transfected with siA_2M and the negative control. (**A**, **B**) Representative western blots and quantification data of A_2M in each group. **P*<0.05.
